# A New Nomenclature for the Very Low-Calorie Ketogenic Diet (VLCKD): Very Low-Energy Ketogenic Therapy (VLEKT). Ketodiets and Nutraceuticals Expert Panels: “KetoNut”, Italian Society of Nutraceuticals (SINut) and the Italian Association of Dietetics and Clinical Nutrition (ADI)

**DOI:** 10.1007/s13668-024-00560-w

**Published:** 2024-07-23

**Authors:** Luigi Barrea, Massimiliano Caprio, Davide Grassi, Arrigo Francesco Giuseppe Cicero, Carmela Bagnato, Barbara Paolini, Giovanna Muscogiuri

**Affiliations:** 1 Dipartimento di Benessere, Nutrizione e Sport, Università Telematica Pegaso, 80143 Naples, Italy; 2 Laboratory of Cardiovascular Endocrinology, IRCCS San Raffaele, Rome, Italy; 3 Department of Human Sciences and Promotion of the Quality of Life, San Raffaele Roma Open University, 00166 Rome, Italy; 4 Department of Life, Health, and Environmental Sciences Via Pompeo Spennati, University of L’Aquila, Viale S Salvatore, Delta 6 Medicina, 67100 Coppito, Italy; 5 Hypertension and Cardiovascular Risk Factors Research Unit, Medical and Surgical Sciences Dept, Alma Mater Studiorum University of Bologna, Via Massarenti 9, 40138 Bologna, Italy; 6 Cardiovascular Medicine Unit, IRCCS AOU Di Bologna, Bologna, Italy; 7 Clinical Nutrition Unit, Madonna Delle Grazie Hospital, Matera, Italy; 8 Italian Association of Dietetics and Clinical Nutrition (Associazione Italiana di Dietetica e Nutrizione Clinica, ADI), Rome, Italy; 9 UOSA of Dietetics and Clinical Nutrition, Azienda Ospedaliera Universitaria Senese, Policlinico Santa Maria alle Scotte, Siena, Italy; 10 Dipartimento di Medicina Clinica e Chirurgia, Unità di Endocrinologia, Diabetologia e Andrologia, Università degli Studi di Napoli Federico II, Via Sergio Pansini 5, 80131 Naples, Italy; 11 Centro Italiano per la cura e il Benessere del paziente con Obesità (C.I.B.O), UOC di Endocrinologia, Diabetologia e Andrologia, Dipartimento di Medicina Clinica e Chirurgia, Unità Di Endocrinologia, Università Degli Studi Di Napoli Federico II Università degli Studi di Napoli Federico II, Naples, Italy; 12 Cattedra Unesco “Educazione alla Salute e allo Sviluppo Sostenibile”, Università Degli Studi Di Napoli Federico II, Naples, Italy; 13 Italian Society of Nutraceuticals (Società Italiana di Nutraceutica, SINut), 40138 Bologna, Italy

**Keywords:** Ketogenic diet, Nomenclature, Very low energy ketogenic therapy, Obesity, Very low calorie ketogenic diets VLCKD

## Abstract

**Purpose of Review:**

In an attempt to clarify the most appropriate nomenclature for the very low-calorie ketogenic diets (VLCKD), we propose to change the nomenclature and acronym of this medical nutrition therapy. The new definition and acronym proposed by the "KetoNut" panel of experts of the Italian Society of Nutraceuticals (SINut) and the Italian Association of Dietetics and Clinical Nutrition (ADI) is Very Low-Energy Ketogenic Therapy (VLEKT).

**Recent Findings:**

In the last few years, different authors have focused on the issue of confusion in the nomenclature of ketogenic diets. In detail, have been differentiated the VLCKD that provides < 800 kcal *per* day, which is intended for the weight loss in the medical treatment of obesity, and a eucaloric ketogenic diet, which contains more calories from fat (predominantly unsaturated) and with specific ketogenic ratios, for allow growth in children while helping, at the same time, to establish epileptic seizure control.

**Summary:**

In recent years, ketogenic diets have attracted great interest for their efficacy in the treatment of epilepsy and other neurological diseases but also in patients with overweight and obesity-related metabolic disorders. Nevertheless, although ketogenic diets are a dietary intervention designed to induce nutritional ketosis, different diets with different macronutrients’ composition have been called with this name. The confusion in the nomenclature of ketogenic diets may result in significant bias and mistakes in the interpretation of the current scientific evidence.

**Graphical Abstract:**

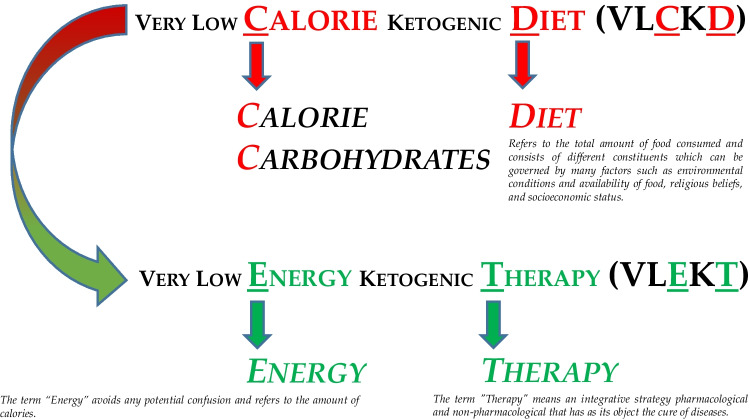

## Introduction

In recent years, ketogenic diets have attracted great interest for their efficacy in the treatment of epilepsy and other neurological diseases but also in patients with overweight and obesity [1]. Differently from epilepsy, where, in order not to interfere with children growth, weight loss during a ketogenic diet is considered one of the side effects to be avoided even in long-term follow-up, the induction of ketosis with ketogenic diets, primarily through calorie and carbohydrate restriction, are primarily intended for weight loss in obesity and its endocrine and metabolic comorbidities [2]. In this context, low carbohydrates diets can be high or moderate in protein and fat and may not necessarily result in nutritional ketosis [3]. Of interest, as some amino acids are used for gluconeogenesis, the amount of protein introduced in the diet appears to influence ketogenesis as well.

However, although ketogenic diet is a dietary intervention designed to induce nutritional ketosis, due to their valuable pleiotropic of effects and heterogeneity, several different diets with different macronutrients’ composition have been called with this name, i.e. the Atkins diet, the classic ketogenic diet, the high-fat ketogenic diet, the very low-calorie ketogenic diet (VLCKD), and the very low-carb ketogenic diet [2].

## The Confusion in the Nomenclature of Ketogenic Diets

The confusion in the nomenclature of ketogenic diets may result in significant bias and mistakes in the interpretation of the current scientific evidence. In the last few years, some Authors have focused on the issue of confusion in the nomenclature of ketogenic diets.

Bistrian B.R. in 2018 differentiated two types of ketogenic diet: the very low–calorie diet that provides < 800 kcal *per* day, which is intended for the weight loss in the medical treatment of obesity and a eucaloric ketogenic diet, contains more calories from fat for allow growth in children while helping to establish seizure control [4].

More recently, an attempt to change the nomenclature of ketogenic diets has been made by Trimboli et al. [5] and by Volek et al. [6] that proposed to define “ketogenic diets” all the diets characterized by a drastic carbohydrate restriction. However, this is not enough to precisely define all the ketogenic diets, mostly because there is the need also to characterize macronutrients’composition and calorie content, which in turn also play a role on the “ketogenic performance”. In particular, proteins display both ketogenic and antiketogenic properties (46–58% respectively) given that an excessive protein intake could stimulate gluconeogenesis thus preventing and/or blunting ketone bodies production. Taken this into account, it appears evident that the percentage of proteins could also a play a major role in ketogenic settings and that their intake should be carefully standardized as well as carbohydrate intake [7, 8].

Therefore, in an attempt to clarify nomenclature, for the very low-calorie diets (< 800 kcal) with reduced carbohydrate content (< 30—50 g) and with a protein content based on the patient's ideal weight, we propose to change the nomenclature and acronym of Very Low Calorie Ketogenic Diets, and adopt the following nomenclature and abbreviation: Very Low Energy Ketogenic Therapy (VLEKT).

## Very Low Energy Ketogenic Therapy (VLEKT)

“Very Low Energy” identifies the amount of daily calorie intake, which is less than 800 kcal therefore referring to the type of ketogenic diet to be used in patients with overweight and obesity in order to achieve rapid weight loss.

The letter "E" of term “Energy” seems preferable than “Calorie” in order to avoid any potential confusion, given that “C” is also the first letter of “carbohydrates” instead of “calorie” [9–11].

As previously reported, it is important to underline that in a ketogenic diet, the only macronutrient that needs to be limited or measured is not just carbohydrates. A common misconception is to consider the ketogenic diet as a high-protein diet: a ketogenic diet, in fact, does not translate to excessively high consumption of animal-based protein. As reported in the recent Ketogenic Nutritional Therapy (KeNuT) SIE-consensus statement [12], a daily protein intake of a minimum of 0.8 g *per* kg of body weight is recommended, and it should not exceed 1.5 g *per* kg of ideal body weight. An excess protein intake in a state of energy restriction will be converted to glucose by gluconeogenesis, and this may have a negative impact on achieving nutritional ketosis. In the context of fats, in order to reach the amounts of lipids prescribed in VLEKT, high fat sources should be part of the dietary plan. This does not mean increasing the quantities of saturated fatty acids since a VLCKD therapy can be elaborated with a high consumption of unsaturated fatty acids. Vegetable oils, for example, rice, wheat germ, and sunflower oils, can be used in addition to extra virgin olive oil. All these oils are rich in polyunsaturated fatty acid (PUFA) and other important compounds with antioxidant and anti-inflammatory action including γ-oryzanol, a bioactive compound with hypolipidemic and antioxidant effects present in rice oil [13]. In addition, as good sources of healthy lipids, proteins, vitamins, minerals, and fiber olives, nuts, and seeds should be consumed daily [14]. Several evidence have shown an inverse correlation between the risk of hyperlipidemia (and cardiovascular disease) and nut consumption [15]. Again, the addition of soy lecithin, commercially available as avocado is a further strategy to increase PUFA intake [16]. Avocado is an optimal source of vitamins, minerals fiber, ω-6 and ω-9, and it contains an oil rich in monounsaturated fatty acid (MUFA), it can used in cream preparation (i.e., guacamole sauce) or be eaten by itself with oil [17]. Avocado fruit has nutritional profile similar to walnuts, pistachios or almonds, which have demonstrate heart health claims [16]. In reverse, mascarpone cheese, butter, cream, and mayonnaise (fats of animal origin) should be consumed in moderation.

The letter “K” of the term “ketogenic” suggests that diet therapy implies an increase in circulating ketone bodies β-hydroxybutyrate (the predominant circulating ‘ketone’), acetoacetate, and acetone (evaluated in blood, urine and breath, respectively) [18].

However, even if ketosis is the most relevant factor to hunger suppression [19], in clinical practice, no target values of ketosis have been identified so far. In general, nutritional ketosis can be defined as a capillary blood β-hydroxybutyrate level of at least 0.5 mmol/L, which should not exceed 5.0 mmol/L [6]. It is essential to underline that these ketone levels are well below ketoacidosis, a pathological condition, which occurs when blood ketones reach concentrations > 25.0 mmol/L [20].

Finally, the letter "T" of term “therapy” highlights that this nutritional protocol should be considered as a valuable nutritional therapy, which requires a preliminary medical evaluation by the physician and a prescription by a qualified nutritionist who can draw up the personalized diet plan, also detailing the hidden sources of carbohydrates to the patient.

Furthermore, the "T" underlines that this dietary therapy, like a drug, has specific indications and contraindications and requires adequate follow-up (medical and nutritional) due to the complex biochemical implications of ketosis [21].

In this scenario, the use of meal replacement should be always taken into account instead of traditional foods as recently proposed by the Italian Society of Endocrinology (SIE), in the consensus statement KeNuT [12].

The use of a multi-step protocol with meal replacements could be useful for calorie control, since most of the patients have the tendency to overestimate the amount of calories in food. In addition, the limited range of flavours of meal replacement could reduce the hedonic drive for food and increase the feeling of satiety compared to natural foods. In the ketosis state Very Low Energy Ketogenic Therapy (VLEKT) is characterized by a very low-energy content (650 – 800 kcal/day), low in carbohydrates (< 30 g daily from vegetables) and fat (only 20 g *per* day, derived from extra virgin olive oil). The amount of high-biological-value protein ranges between 0.8 and 1.2 g *per* each kg of ideal body weight. After the ketogenic state, there is a low-calorie, non-ketogenic state in which there is a progressive and slow reintroduction of different food groups, starting from foods with the lowest glycaemic index (first fruit, then dairy products, subsequently legumes) to foods with higher glycemic index (cereals). Generally, the amount of carbohydrate *per* day in low-carbohydrate diets is approximately 60 – 130 g (≤ 20–45% of daily energy intake). Once the reintroduction of all food classes has been completed, a hypocaloric Mediterranean diet is restored tailoring energy intake according to individual characteristics (age, weight, height, climate, physical activity, etc.) [12].

## Conclusions

**Fig. 1:**
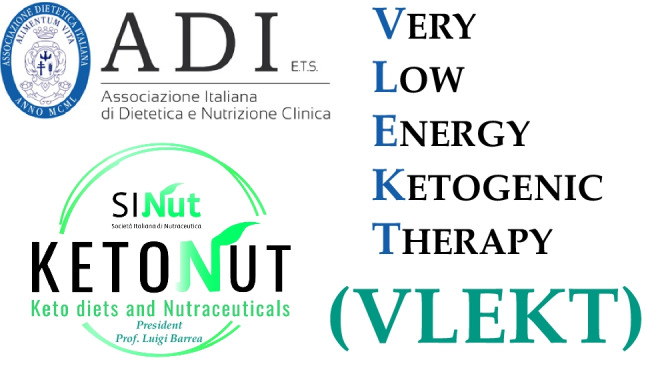
A New Nomenclature for the Very Low-Calorie Ketogenic Diet (VLCKD): Very Low-Energy Ketogenic Therapy (VLEKT): Ketodiets and Nutraceuticals Expert Panels: “KetoNut”, Italian Society of Nutraceuticals (SINut) and the Italian Association of Dietetics and Clinical Nutrition (ADI)

The new definition and acronym Very Low-Energy Ketogenic Therapy (VLEKT) proposed by the "KetoNut" panel of experts of the Italian Society of Nutraceuticals (SINut) and the Italian Association of Dietetics and Clinical Nutrition (ADI), aims at standardizing the nomenclature of very low-calorie ketogenic diets (VLCKD) in patients with obesity, in the attempt to contribute for a more homogeneous and interpretable body of evidence related to clinical trials with ketogenic diets; Fig. 1.

## Declarations

### Copyright for the figures

The figure and graphic abstract that were included in the manuscript are original. The use of the logos of the two scientific societies has been authorized by the respective presidents, Prof. Arrigo Francesco Giuseppe Cicero (SINut President) and Barbara Paolini (ADI President).

## References

[1] Muscogiuri G, El Ghoch M, Colao A, Hassapidou M, Yumuk V, Busetto L. European Guidelines for Obesity Management in Adults with a Very Low-Calorie Ketogenic Diet: A Systematic Review and Meta-Analysis. Obes Facts. 2021;14(2):222–45. 10.1159/000515381. 33882506 10.1159/000515381 PMC8138199

[2] Tagliabue A, Armeno M, Berk KA, Guglielmetti M, Ferraris C, Olieman J, et al. Ketogenic diet for epilepsy and obesity is it the same. Nutr Metab Cardiovasc Dis. 2024;34:581–9. http://www.ncbi.nlm.nih.gov/pubmed/38326186. 38326186 10.1016/j.numecd.2024.01.014

[3] Kirkpatrick CF, Bolick JP, Kris-Etherton PM, Sikand G, Aspry KE, Soffer DE, et al. Review of current evidence and clinical recommendations on the effects of low-carbohydrate and very-low-carbohydrate (including ketogenic) diets for the management of body weight and other cardiometabolic risk factors: A scientific statement from the Nati. J Clin Lipidol. 2019;13:689-711.e1. http://www.ncbi.nlm.nih.gov/pubmed/31611148. 31611148 10.1016/j.jacl.2019.08.003

[4] Bistrian BR. Two Types of Very Low-Carbohydrate Diets. Pediatrics. 2018;142(2):e20181536A. 10.1542/peds.2018-1536A. 30065006 10.1542/peds.2018-1536A

[5] Trimboli P, Castellana M, Bellido D, Casanueva FF. Confusion in the nomenclature of ketogenic diets blurs evidence. Rev Endocr Metab Disord. 2020;21(1):1–3. 10.1007/s11154-020-09546-9. 32080796 10.1007/s11154-020-09546-9

[6] Volek JS, Phinney SD, Krauss RM, Johnson RJ, Saslow LR, Gower B, et al. Alternative Dietary Patterns for Americans: Low-Carbohydrate Diets. Nutrients. 2021;13(10):3299–321. 10.3390/nu13103299. 34684300 10.3390/nu13103299 PMC8537012

[7] Zilberter T, Zilberter Y. Ketogenic Ratio Determines Metabolic Effects of Macronutrients and Prevents Interpretive Bias. Front Nutr. 2018;5:75–82. 10.3389/fnut.2018.00075. 30214902 10.3389/fnut.2018.00075 PMC6125377

[8] Szendi K, Murányi E, Hunter N, Németh B. Methodological Challenges and Confounders in Research on the Effects of Ketogenic Diets: A Literature Review of Meta-Analyses. Foods. 2024;13(2):248–64. 10.3390/foods13020248. 38254549 10.3390/foods13020248 PMC10814162

[9] Bueno NB, de Melo ISV, de Oliveira SL, da Rocha Ataide T. Very low carbohydrate ketogenic diet v low fat diet for long term weight loss a meta-analysis of randomised controlled trials. Br J Nutr. 2013;110:1178–87. http://www.ncbi.nlm.nih.gov/pubmed/23651522. 23651522 10.1017/S0007114513000548

[10] Rafiullah M, Musambil M, David SK. Effect of a very low-carbohydrate ketogenic diet vs recommended diets in patients with type 2 diabetes a meta-analysis. Nutr Rev. 2022;80:488–502. http://www.ncbi.nlm.nih.gov/pubmed/34338787. 34338787 10.1093/nutrit/nuab040

[11] Tzenios N, Lewis ED, Crowley DC, Chahine M, Evans M. Examining the Efficacy of a Very-Low-Carbohydrate Ketogenic Diet on Cardiovascular Health in Adults with Mildly Elevated Low Density Lipoprotein Cholesterol in an Open-Label Pilot Study. Metab Syndr Relat Disord. 2022;20:94–103. http://www.ncbi.nlm.nih.gov/pubmed/34918971. 34918971 10.1089/met.2021.0042 PMC8972001

[12] Barrea L, Caprio M, Camajani E, Verde L, Perrini S, Cignarelli A, et al. Ketogenic nutritional therapy (KeNuT)-a multi-step dietary model with meal replacements for the management of obesity and its related metabolic disorders: a consensus statement from the working group of the Club of the Italian Society of Endocrinology. J Endocrinol Invest. 2024;47:487–500. http://www.ncbi.nlm.nih.gov/pubmed/38238506. 38238506 10.1007/s40618-023-02258-2 PMC10904420

[13] Ramazani E, Akaberi M, Emami SA, Tayarani-Najaran Z. Biological and Pharmacological Effects of Gamma oryzanol An Updated Review of the Molecular Mechanisms. Curr Pharm Des. 2021;27:2299–316. http://www.ncbi.nlm.nih.gov/pubmed/33138751 33138751 10.2174/1381612826666201102101428

[14] Qamar S, Manrique YJ, Parekh H, Falconer JR. Nuts, cereals, seeds and legumes proteins derived emulsifiers as a source of plant protein beverages. Crit Rev Food Sci Nutr. 2020;60:2742–62. http://www.ncbi.nlm.nih.gov/pubmed/31478387. 31478387 10.1080/10408398.2019.1657062

[15] Balakrishna R, Bjørnerud T, Bemanian M, Aune D, Fadnes LT. Consumption of Nuts and Seeds and Health Outcomes Including Cardiovascular Disease, Diabetes and Metabolic Disease, Cancer, and Mortality an Umbrella Review. Advances in nutrition. 2022;16:2136–48. http://www.ncbi.nlm.nih.gov/pubmed/36041171. 10.1093/advances/nmac077 PMC9776667 36041171

[16] Okobi OE, Odoma VA, Okunromade O, Louise-Oluwasanmi O, Itua B, Ndubuisi C, et al. Effect of Avocado Consumption on Risk Factors of Cardiovascular Diseases a Systematic Review and Meta Analysis. Cureus. 2023;15:e41189. http://www.ncbi.nlm.nih.gov/pubmed/37525782. 37525782 10.7759/cureus.41189 PMC10387226

[17] Unlu NZ, Bohn T, Clinton SK, Schwartz SJ. Carotenoid absorption from salad and salsa by humans is enhanced by the addition of avocado or avocado oil. J Nutr. 2005;135:431–6. http://www.ncbi.nlm.nih.gov/pubmed/15735074. 15735074 10.1093/jn/135.3.431

[18] van Delft R, Lambrechts D, Verschuure P, Hulsman J, Majoie M. Blood beta-hydroxybutyrate correlates better with seizure reduction due to ketogenic diet than do ketones in the urine. Seizure. 2010;19:36–9;19:36–9. http://www.ncbi.nlm.nih.gov/pubmed/19962324. 19962324 10.1016/j.seizure.2009.10.009

[19] Gibson AA, Seimon RV, Lee CMY, Ayre J, Franklin J, Markovic TP, et al. Do ketogenic diets really suppress appetite? A systematic review and meta-analysis. Obes Rev. 2015;16(1):64–76. 10.1111/obr.12230. 25402637 10.1111/obr.12230

[20] Koppel SJ, Swerdlow RH. Neuroketotherapeutics A modern review of a century-old therapy. Neurochem Int. 2018;117:114–25. http://www.ncbi.nlm.nih.gov/pubmed/28579059. 28579059 10.1016/j.neuint.2017.05.019 PMC5711637

[21] Caprio M, Infante M, Moriconi E, Armani A, Fabbri A, Mantovani G, et al. Very-low-calorie ketogenic diet (VLCKD) in the management of metabolic diseases: systematic review and consensus statement from the Italian Society of Endocrinology (SIE). J Endocrinol Invest. 2019;42(11):1365–86. 10.1007/s40618-019-01061-2. 31111407 10.1007/s40618-019-01061-2

